# Glutathione S-transferase M1 and T1 polymorphisms in Arab glaucoma patients

**Published:** 2008-03-04

**Authors:** Khaled K. Abu-Amero, Jose Morales, Gamal H. Mohamed, Mazen N. Osman, Thomas M. Bosley

**Affiliations:** 1 Genetics Department, King Faisal Specialist Hospital and Research Centre, Riyadh. Saudi Arabia; 2 Department of Biostatistics, Epidemiology and Scientific Computing, King Faisal Specialist Hospital and Research Centre, Riyadh. Saudi Arabia; 3 Neuroscience Department, King Faisal Specialist Hospital and Research Centre, Riyadh; 4 Glaucoma Division, King Khaled Eye Specialist Hospital, Riyadh, Saudi Arabia; 5 Neuro-ophthalmology Division, King Khaled Eye Specialist Hospital, Riyadh, Saudi Arabia

## Abstract

**Purpose:**

Glutathione S-transferases (GSTs) are a family of enzymes that inactivate xenobiotics and endogenous end products formed as secondary metabolites during oxidative stress. In humans, *GSTT1* and *GSTM1* deletion genotypes (T0M1, T1M0, and T0M0) are associated with a variety of pathologic processes including certain ophthalmologic diseases.

**Methods:**

We compared the prevalence of *GSTT1* and *GSTM1* deletion genotypes, which were determined by multiplex polymerase chain reaction, in 107 Arab patients with glaucoma (49 with primary open-angle glaucoma, 29 with pseudoexfoliation glaucoma, and 29 with primary angle-closure glaucoma) to 120 age, sex, and ethnically matched controls.

**Results:**

All three GST polymorphisms were significantly more common in the entire glaucoma group (p<0.0167) than in controls. However, when patients were stratified by glaucoma type, the deletion genotype, T0M0, was not particularly associated with any type of glaucoma tested. The T1MO genotype was more common among patients with each type of glaucoma than among controls whereas T0M1 genotype was more common among pseudoexfoliation glaucoma (PEG) and primary open-angle glaucoma (POAG) patients than controls.

**Conclusions:**

The overall results indicate a possible variable association between various *GSTT1* and *GSTM1* genotypes and glaucoma in this population. Decreased GST function might interfere with the metabolism of oxidative intermediates and exacerbate the direct or indirect damaging effects of oxidative stress on the optic nerve. It is possible that these GST polymorphisms may be risk factors for glaucoma.

## Introduction

Glaucoma is the most common optic neuropathic process affecting humans and the second most common cause of blindness worldwide [1]. It is a complex, heterogeneous disease with a multifactorial etiology including mechanical damage due to increased intraocular pressure (IOP), variable susceptibility of the optic nerve [2], mutations in specific nuclear genes [3], changes in the mitochondrial genome [4], and toxic effects and oxidative damage caused by reactive oxygen species (ROS) [3,5,6].

The glutathione S-transferases (GSTs) are a family of enzymes consisting of numerous cytosolic, mitochondrial, and microsomal proteins capable of multiple reactions with endogenous and xenobiotic substrates. They catalyze the conjugation of reduced glutathione to electrophilic centers via the sulfhydryl group on a wide variety of substrates [7]. GSTs bind toxins, function as transport proteins, detoxify endogenous compounds such as peroxidized lipids [8], and inactivate endogenous end products formed as secondary metabolites during oxidative stress.

In humans, GST enzymes are divided into five classes − alpha (A), mu (M), pi (P), theta (T) and zeta (Z) − with each class encompassing several genes and isoenzymes. Previous studies of allelic variants in these classes have identified two major polymorphisms of the *GSTT1* and *GSTM1* genes caused by a deletion in each gene. These deletions result in the virtual absence of enzyme activity, particularly in individuals with deletions in both genes (null genotype). The null genotype (T0M0) has been associated with altered risk of a variety of pathologies including cancer, cardiovascular disease, and respiratory disease [9-11]. GST polymorphisms have also been associated with ophthalmologic problems such as cataract [12] and senile macular degeneration [13].

We recently reported evidence of mitochondrial abnormalities in patients with primary open-angle glaucoma (POAG), implying that an oxidative stress mechanism might contribute to the pathogenesis of POAG [4]. In fact, several studies have found GST polymorphisms to be risk factors in POAG [14-17]. Because of the role of GSTs in inactivating endogenous end products formed as secondary metabolites during oxidative stress, we decided to compare the distribution of *GSTM1* and *GSTT1* polymorphisms in Saudi patients with POAG, pseudoexfoliation glaucoma (PEG), and primary angle-closure glaucoma (PACG) to the distribution in matching healthy controls.

## Methods

### Patients and controls

A total of 107 glaucoma patients (49 with POAG, 29 with PEG, and 29 with PACG) were enrolled in this study. The inclusion and exclusion criteria for each type of glaucoma were detailed elsewhere [4,18,19]. All patients were Saudi Arabs selected from the Glaucoma Clinic at King Khaled Eye Specialist Hospital (KKESH) after an examination by a glaucoma specialist (J.M.) and informed consent approved by the KKESH IRB. Records were reviewed, a general medical history was taken, and complete ophthalmologic examinations were performed. Patients had either Goldmann manual kinetic perimetry (Haag Streit AG, Koeniz-Berne, Switzerland), Humphrey automated white on white stimulus static perimetry (Humphrey Field Analyzer II, Humphrey Systems, Dublin, CA), or both. Optical coherence tomography was performed with the OCT3 Unit by Humphrey Systems (San Leandro, CA) on some patients. Fundus photos were obtained using a Zeiss FF 450 system and conventional film. This research followed the tenets of the Declaration of Helsinki. Family members were not evaluated clinically or genetically.

Control subjects were blood donors at the King Faisal Specialist Hospital and Research Centre who represented the spectrum of Saudi Arabs and who reported no symptomatic, metabolic, genetic, or ocular disorder on an extensive questionnaire about family history, past medical problems, and current health.

### Sample collection and DNA extraction

Peripheral blood (5 ml) was collected in EDTA tubes from all participating individuals after obtaining their written consent. DNA extraction was performed using the PURGENE DNA isolation kit from Gentra Systems (Minneapolis, MN), and the DNA was stored in aliquots at –20 °C until required.

### Analysis of *GSTM1* and *GSTT1* polymorphisms

This was performed by multiplex polymerase chain reaction (PCR) amplification using the following primers: *GSTT1* forward primer 5`-TTC CTT ACT GGT CCT CAC ATC TC −3`; *GSTT1* reverse primer 5`-TCA CCG GAT CAT GGC CAG CA −3`; *GSTM1* forward primer 5`- GAA CTC CCT GAA AAG CTA AAG C −3`; and *GSTM1* reverse primer 5`- GTT GGG CTC AAA TAT ACG GTG G −3`. Each 25 µl PCR reaction contained 2.5 µl of 10X reaction buffer with MgCl_2_ (Amersham Pharmacia Biotech, Piscataway, NJ), 10 ρmol of each primer, 100 ρmol/µl each of deoxynucleoside triphosphates (deoxyATP, deoxyguanosine triphosphate, deoxycytidine triphosphate, and deoxythymidine triphosphate; Perkin-Elmer Corporation, Foster City, CA) in Tris-HCl buffer, 1 unit of HotStar Taq DNA polymerase (Amersham Pharmacia Biotech, Piscataway, NJ), and 100 ng genomic DNA template at annealing temperature of 58 °C for 40 cycles. The PCR products were visualized on a 2% agarose gel electrophoresis at 100 V for 50 min. Two bands of 459 bp for *GSTT1* and 209 bp for *GSTM1* were obtained for the T1M1 genotype. The T1M0 genotype showed one band of 459 bp, and the T0M1 genotype showed a band of 209 bp. For the T0M0 genotype (homozygous absence or deletion genotype is designated as null genotype), no bands were obtained and thus the use of β-globin internal positive control was necessary to distinguish the null genotype from aborted PCR reactions.

### Statistical analysis

Genotype frequencies, sex distribution, and smoking history were compared among the different glaucoma groups and the controls by the chi square test. The mean age comparison between patients and controls was analyzed by *t*-test. A two-tailed p value of less than 0.05 was considered statistically significant, and odds ratio with 95% confidence intervals were reported. The Bonferroni correction was used to adjust the significance level of a statistical test to protect against Type I errors when multiple comparisons are being made. All analyses were performed using SPSS v.13 statistical analysis software (SPSS Inc., Chicago, IL).

## Results

Most of these patients were included in previous reports of nuclear and mitochondrial genetics in patients with glaucoma [4,18,19]. The control group consisted of 120 individuals (78 males and 42 females, mean age 60.5 ± 9.1 years). All patients and control subjects were of Saudi Arabian origin.

Table 1 shows demographic data for glaucoma patients and controls. Patients and controls were not significantly different with respect to sex, age, and smoking status (p>0.0167); therefore, these variables were not evaluated further.

**Table 1:** Demographic data of the study groups.

**Study group**	**Glaucoma group**	**Control group**
**Number of subjects**	107	120

**Sex**		
Male, n (%)	68 (63.6%)	78 (65.0%)
Female, n (%)	39 (36.4%)	42 (35.0%)

**Age (years)**		
Mean (SD)	60.6 (8.8)	60.5 (9.1)

**Smoking**		
Yes, n (%)	5 (4.7%)	7 (5.8%)
No, n (%)	95 (88.8%)	113 (94.2%)
Unknown, n (%)	7 (6.5%)	0

Patients and controls were not significantly different with respect to sex, age and smoking status (p>0.0167).

Table 2 shows the GST genotype distribution among all glaucoma patients and controls. The frequency of all genotypes investigated was significantly greater among glaucoma patients than controls.

**Table 2:** Glutathione S-transferase genotypes and the risk of developing glaucoma.

**GST genotypes**	**Controls (n=120)**	**Glaucoma patients (n=107)**	**Odds ratio**	**95% Confidence interval**	**p-Value**
T1M1	102 (85%)	43 (40.2%)	Reference	-	-
T0M0	3 (2.5%)	9 (8.4%)	7.12	1.65 – 35.0	0.002
T1M0	10 (8.3%)	38 (35.5%)	9.01	3.89 – 21.3	0.00001
T0M1	5 (4.2%)	17 (15.9%)	8.07	2.58 – 26.9	0.00001

The frequency of all deletion genotypes investigated was significantly greater among glaucoma patients than controls (after Bonferroni correction, p-value < 0.0167 was considered statistically significant).

We also examined GST genotype distribution in all three variants of glaucoma. Table 3 shows that the frequency of the T1M0 and T0M1 genotypes was significantly greater in POAG patients than in controls (p=0.00001) while the frequency of the T0M0 null genotype was not significant (p=0.06). Table 4 shows that all deletion GST genotype frequencies were greater in PEG patients than in controls, except for the T0M0 null genotype, which fell slightly short of significance (p=0.021). Table 5 shows that the frequency of the T1M0 genotypes was significantly greater in PACG patients than in controls (p=0.0002) whereas T0M0 and T0M1 were not significant.

**Table 3:** Glutathione S-transferase genotypes and the risk of developing primary open-angle glaucoma.

**GST genotypes**	**Controls (n=120)**	**POAG patients (n=49)**	**Odds ratio**	**95% Confidence interval**	**p-Value**
T1M1	102 (85%)	18(36.7%)	Reference	-	-
T0M0	3 (2.5%)	3 (6.2%)	5.67	0.83 – 39.2	0.06
T1M0	10 (8.3%)	18 (36.7%)	10.2	3.72 – 28.6	0.00001
T0M1	5 (4.2%)	10 (20.4%)	11.3	3.07 – 44.0	0.00001

The frequency of the T1M0 and T0M1 genotypes was significantly greater in POAG patients than controls (p=0.00001), while the frequency of the T0M0 null genotype which was not significant (p=0.06). After Bonferroni correction, p-value < 0.0167 was considered statistically significant.

**Table 4:** Glutathione S-transferase genotypes and the risk of developing pseudoexfoliation glaucoma.

**GST genotypes**	**Controls (n=120)**	**PEG patients (n=29)**	**Odds ratio**	**95% Confidence interval**	**p-Value**
T1M1	102 (85%)	11 (37.9%)	reference	-	-
T0M0	3 (2.5%)	3 (10.3%)	9.27	1.28 – 68.5	0.021
T1M0	10 (8.3%)	10 (34.5%)	9.27	2.81 – 31.3	0.00001
T0M1	5 (4.2%)	5 (17.3%)	9.27	1.93 – 45.8	0.003

All deletion GST genotype frequencies were greater in PEG patients than controls, except for the T0M0 null genotype, which fell slightly short of significance (p=0.021). After Bonferroni correction, p-value < 0.0167 was considered statistically significant.

**Table 5:** Glutathione S-transferase genotypes and the risk of developing primary angle-closure.

**GST genotypes**	**Controls (n=120)**	**PACG patients (n=29)**	**Odds ratio**	**95% Confidence interval**	**p-Value**
T1M1	102 (85%)	14 (48.3%)	Reference	-	-
T0M0	3 (2.5%)	3 (10.3%)	7.29	1.04 – 51.9	0.034
T1M0	10 (8.3%)	10 (34.5%)	7.29	2.30 – 23.5	0.0002
T0M1	5 (4.2%)	2 (6.9%)	2.91	0.35 – 19.7	0.225

The frequency of the T1M0 genotype was significantly greater in PACG patients than in controls (p=0.0002), whereas the prevalence of T0M0 and T0M1 genotypes were not statistically different from controls. After Bonferroni correction, p-value < 0.0167 was considered statistically significant.

## Discussion

Glaucoma patients in this study met strict criteria for POAG, PEG, and PACG, which have been described previously [4,18,19]. Controls in this study were well matched to patients for age, sex, ethnicity, and smoking status. The T1M0 and the T0M1 genotypes were significantly more common in the entire glaucoma group and were almost all more common in each individual variant of glaucoma as well. The full deletion (T0M0 null genotype) was almost not associated with any particular type of glaucoma. We believe that this is the first study to quantify the prevalence of these polymorphisms among Arab patients with various types of glaucoma.

Our results are in concordance with previous studies associating *GSTT1* or *GSTM1* gene deletions with POAG in Turkish [14,17], Estonian [15], and Italian [16] populations, although one study reported no association between these genotypes and POAG in a Swedish population [20]. The lack of association found in the Swedish population may represent a population specific effect, e.g., caused by differences in genetic background between various world populations. The frequency of these genotypes among controls reported here was similar to that reported previously in Saudi Arabs [9] but moderately different from frequencies reported in a Turkish population [17]. These differences may reflect varying ethnic and genetic backgrounds of the populations studied. We were unable to determine whether these polymorphisms were in Hardy–Weinberg equilibrium because heterozygous individuals could not be distinguished from homozygous wild type and because the high consanguinity rate in the Saudi population (>65%) means that the random mating requirement is not satisfied [21]. We did not quantify the interaction between GST genotypes, smoking status, and the development of glaucoma, since only five of our glaucoma patients (4.7%) were smokers.

Glaucoma is a heterogeneous disease with several important variants that differ at least partially in the apparent mechanisms of optic nerve injury. For example, POAG typically has lower IOP and no obvious ocular changes (in contrast to PEG or PACG) and is suspected of being associated with molecular and biochemical abnormalities [2]. Optic nerve injury in PEG is a component of pseudoexfoliation syndrome, a systemic condition [22,23] that may include stroke and myocardial infarction [24,25], and is frequently associated with severe chronic open-angle glaucoma and cataract [26]. A recent genome-wide screen of Icelandic and Swedish populations detected an association of PEG with two nonsynonymous single nucleotide polymorphisms (SNPs) in exon 1 of the *LOXL1* gene [27]. Unfortunately, samples were no longer available for us to test this association among our PEG patients.

PACG has been attributed primarily to elevated IOP caused by anatomic changes in the anterior [28] and posterior [29] globe. However, in each variant, ocular phenomena must interact with the posterior globe to cause optic nerve injury [26,29,30], and recent evidence supports the hypothesis that ROS and oxidative stress may play a contributing role in glaucomatous optic nerve injury at several levels [31]. Oxidative stress may be directly involved in optic nerve neuronal cell death [32]. In addition, human trabecular meshwork (TM) possesses abundant antioxidant activity, and ROS compromise TM integrity [16,33]. Oxidative DNA damage is increased in TM in POAG [34], which might induce degenerative changes favoring increased IOP [34]. Therefore, oxidative stress early in the development and/or throughout life might precipitate both metabolic and anatomic sequelae that increase the risk of optic nerve damage in glaucoma. This may be true even in glaucoma variants that seem primarily anatomic in the mechanism of optic nerve injury such as PACG.

GST enzymes inactivate endogenous unsaturated aldehydes, quinines, epoxides, and hydroperoxides and also protect blood vessels from the effects of endogenous oxidants formed as secondary metabolites during oxidative stress [35]. It is plausible that decreased GST enzyme activity might contribute to inadequate inactivation of these metabolites, exacerbating oxidative stress and increasing the chance of glaucomatous optic nerve injury directly (e.g., in the optic nerve) or remotely (e.g., in the TM and possibly elsewhere in the globe) or both. These effects might be independent of the specific ocular characteristics of the glaucoma syndrome. In fact, the results presented here imply that defects in GST activity may well be risk factors for developing all three variants of glaucoma evaluated. The exact mechanisms by which this occurs are not clear, which is not surprising given that the exact mechanisms of GST activity have yet to be elucidated [35].

In conclusion, this study indicates an association of *GSTT1* and *GSTM1* polymorphisms to a variable degree with three types of glaucoma. Further investigations are warranted into the precise mechanism by which these genetic polymorphisms may influence the development of glaucoma.

## References

[1] Quigley HA (1996). Number of people with glaucoma worldwide.. Br J Ophthalmol.

[2] Epstein DL. Primary open angle glaucoma. In: Epstein DL, Allingham RR, Schuman JS, editors. Chandler and Grant's Glaucoma. 4th ed. Baltimore: Williams & Wilkins; 1997. p. 183–98.

[3] Wiggs JL (2007). Genetic etiologies of glaucoma.. Arch Ophthalmol.

[4] Abu-Amero KK, Morales J, Bosley TM (2006). Mitochondrial abnormalities in patients with primary open-angle glaucoma.. Invest Ophthalmol Vis Sci.

[5] Liu Q, Ju WK, Crowston JG, Xie F, Perry G, Smith MA, Lindsey JD, Weinreb RN (2007). Oxidative stress is an early event in hydrostatic pressure induced retinal ganglion cell damage.. Invest Ophthalmol Vis Sci.

[6] Tezel G (2006). Oxidative stress in glaucomatous neurodegeneration: mechanisms and consequences.. Prog Retin Eye Res.

[7] Boyer TD (1989). The glutathione S-transferases: an update.. Hepatology.

[8] Tocher DR, Leaver MJ, Hodgson PA (1998). Recent advances in the biochemistry and molecular biology of fatty acyl desaturases.. Prog Lipid Res.

[9] Abu-Amero KK, Al-Boudari OM, Mohamed GH, Dzimiri N (2006). T null and M null genotypes of the glutathione S-transferase gene are risk factor for CAD independent of smoking.. BMC Med Genet.

[10] Habdous M, Siest G, Herbeth B, Vincent-Viry M, Visvikis S (2004). Glutathione S-transferases genetic polymorphisms and human diseases: overview of epidemiological studies.. Ann Biol Clin (Paris).

[11] Strange RC, Spiteri MA, Ramachandran S, Fryer AA (2001). Glutathione-S-transferase family of enzymes.. Mutat Res.

[12] Sekine Y, Hommura S, Harada S (1995). Frequency of glutathione-S-transferase 1 gene deletion and its possible correlation with cataract formation.. Exp Eye Res.

[13] Oz O, Aras Ates N, Tamer L, Yildirim O, Adiguzel U (2006). Glutathione S-transferase M1, T1, and P1 gene polymorphism in exudative age-related macular degeneration: a preliminary report.. Eur J Ophthalmol.

[14] Yildirim O, Ates NA, Tamer L, Oz O, Yilmaz A, Atik U, Camdeviren H (2005). May glutathione S-transferase M1 positive genotype afford protection against primary open-angle glaucoma?. Graefes Arch Clin Exp Ophthalmol.

[15] Juronen E, Tasa G, Veromann S, Parts L, Tiidla A, Pulges R, Panov A, Soovere L, Koka K, Mikelsaar AV (2000). Polymorphic glutathione S-transferase M1 is a risk factor of primary open-angle glaucoma among Estonians.. Exp Eye Res.

[16] Izzotti A, Sacca SC, Cartiglia C, De Flora S (2003). Oxidative deoxyribonucleic acid damage in the eyes of glaucoma patients.. Am J Med.

[17] Unal M, Guven M, Devranoglu K, Ozaydin A, Batar B, Tamcelik N, Gorgon EE, Ucar D, Sarici A (2007). Glutathione S transferase M1 and T1 genetic polymorphisms are related to the risk of primary open-angle glaucoma: a study in a Turkish population.. Br J Ophthalmol.

[18] Abu-Amero KK, Bosley TM, Morales J (2008). Analysis of Nuclear and Mitochondrial Genes in Patients with Pseudoexfoliation Glaucoma.. Mol Vis.

[19] Abu-Amero KK, Morales J, Osman MN, Bosley TM (2007). Nuclear and Mitochondrial Analysis of Patients with Primary Angle Closure Glaucoma.. Invest Ophthalmol Vis Sci.

[20] Jansson M, Rada A, Tomic L, Larsson LI, Wadelius C (2003). Analysis of the Glutathione S-transferase M1 gene using pyrosequencing and multiplex PCR–no evidence of association to glaucoma.. Exp Eye Res.

[21] Panter-Brick C (1991). Parental responses to consanguinity and genetic disease in Saudi Arabia.. Soc Sci Med.

[22] Schlotzer-Schrehardt UM, Koca MR, Naumann GO, Volkholz H (1992). Pseudoexfoliation syndrome. Ocular manifestation of a systemic disorder?. Arch Ophthalmol.

[23] Streeten BW, Li ZY, Wallace RN, Eagle RC, Keshgegian AA (1992). Pseudoexfoliative fibrillopathy in visceral organs of a patient with pseudoexfoliation syndrome.. Arch Ophthalmol.

[24] Mitchell P, Wang JJ, Smith W (1997). Association of pseudoexfoliation syndrome with increased vascular risk.. Am J Ophthalmol.

[25] Repo LP, Terasvirta ME, Koivisto KJ (1993). Generalized transluminance of the iris and the frequency of the pseudoexfoliation syndrome in the eyes of transient ischemic attack patients.. Ophthalmology.

[26] Schlotzer-Schrehardt U, Naumann GO (2006). Ocular and Systemic Pseudoexfoliation Syndrome.. Am J Ophthalmol.

[27] Thorleifsson G, Magnusson KP, Sulem P, Walters GB, Gudbjartsson DF, Stefansson H, Jonsson T, Jonasdottir A, Jonasdottir A, Stefansdottir G, Masson G, Hardarson GA, Petursson H, Arnarsson A, Motallebipour M, Wallerman O, Wadelius C, Gulcher JR, Thorsteinsdottir U, Kong A, Jonasson F, Stefansson K (2007). Common Sequence Variants in the LOXL1 Gene Confer Susceptibility to Exfoliation Glaucoma.. Science.

[28] Ritch R, Lowe RF. Angle-Closure Glaucoma: Mechanisms and Epidemiology. In: Ritch R, Shields MB, Krupin T, editors. The Glaucomas. 2nd ed. St. Louis: Mosby; 1996. p. 801–19.

[29] Quigley HA, Friedman DS, Congdon NG (2003). Possible mechanisms of primary angle-closure and malignant glaucoma.. J Glaucoma.

[30] Pache M, Flammer J (2006). Sick eye in a sick body: Systemic findings in patients with primary open angle glaucoma.. Surv Ophthalmol.

[31] Izzotti A, Bagnis A, Sacca SC (2006). The role of oxidative stress in glaucoma.. Mutat Res.

[32] Osborne NN, Chidlow G, Layton CJ, Wood JP, Casson RJ, Melena J (2004). Optic nerve and neuroprotection strategies.. Eye.

[33] Sacca SC, Izzotti A, Rossi P, Traverso C (2007). Glaucomatous outflow pathway and oxidative stress.. Exp Eye Res.

[34] Sacca SC, Pascotto A, Camicione P, Capris P, Izzotti A (2005). Oxidative DNA damage in the human trabecular meshwork: clinical correlation in patients with primary open-angle glaucoma.. Arch Ophthalmol.

[35] Hayes JD, Flanagan JU, Jowsey IR (2005). Glutathione transferases.. Annu Rev Pharmacol Toxicol.

